# Anesthetic Propofol Reduces Endotoxic Inflammation by Inhibiting Reactive Oxygen Species-regulated Akt/IKKβ/NF-κB Signaling

**DOI:** 10.1371/journal.pone.0017598

**Published:** 2011-03-08

**Authors:** Chung-Hsi Hsing, Ming-Chung Lin, Pui-Ching Choi, Wei-Ching Huang, Jui-In Kai, Cheng-Chieh Tsai, Yi-Lin Cheng, Chia-Yuan Hsieh, Chi-Yun Wang, Yu-Ping Chang, Yu-Hong Chen, Chia-Ling Chen, Chiou-Feng Lin

**Affiliations:** 1 Department of Anesthesiology, Chi Mei Medical Center, Tainan, Taiwan; 2 Department of Anesthesiology, College of Medicine, Taipei Medical University, Taipei, Taiwan; 3 Institute of Clinical Medicine, College of Medicine, National Cheng Kung University, Tainan, Taiwan; 4 Institute of Basic Medical Sciences, College of Medicine, National Cheng Kung University, Tainan, Taiwan; 5 Department of Nursing, Chung Hwa University of Medical Technology, Tainan, Taiwan; 6 Department of Medical Laboratory Science and Biotechnology, College of Medicine, National Cheng Kung University, Tainan, Taiwan; 7 Department of Microbiology and Immunology, College of Medicine, National Cheng Kung University, Tainan, Taiwan; Fundação Oswaldo Cruz, Brazil

## Abstract

**Background:**

Anesthetic propofol has immunomodulatory effects, particularly in the area of anti-inflammation. Bacterial endotoxin lipopolysaccharide (LPS) induces inflammation through toll-like receptor (TLR) 4 signaling. We investigated the molecular actions of propofol against LPS/TLR4-induced inflammatory activation in murine RAW264.7 macrophages.

**Methodology/Principal Findings:**

Non-cytotoxic levels of propofol reduced LPS-induced inducible nitric oxide synthase (iNOS) and NO as determined by western blotting and the Griess reaction, respectively. Propofol also reduced the production of tumor necrosis factor-α (TNF-α), interleukin (IL)-6, and IL-10 as detected by enzyme-linked immunosorbent assays. Western blot analysis showed propofol inhibited LPS-induced activation and phosphorylation of IKKβ (Ser180) and nuclear factor (NF)-κB (Ser536); the subsequent nuclear translocation of NF-κB p65 was also reduced. Additionally, propofol inhibited LPS-induced Akt activation and phosphorylation (Ser473) partly by reducing reactive oxygen species (ROS) generation; inter-regulation that ROS regulated Akt followed by NF-κB activation was found to be crucial for LPS-induced inflammatory responses in macrophages. An *in vivo* study using C57BL/6 mice also demonstrated the anti-inflammatory properties against LPS in peritoneal macrophages.

**Conclusions/Significance:**

These results suggest that propofol reduces LPS-induced inflammatory responses in macrophages by inhibiting the interconnected ROS/Akt/IKKβ/NF-κB signaling pathways.

## Introduction

Propofol (2,6-diisopropylphenol) was originally described as an anesthetic and is routinely used for the short-term, humans sedation in surgery as well as in combined treatments for patients with critical illnesses. Propofol produces a variety of pharmacodynamic effects, ranging from hypnosis to general anesthesia; it is also an excellent amnestic and muscle relaxant [1]. In addition to its pharmacological properties, propofol also exhibits immunomodulatory effects by decreasing the production of pro-inflammatory cytokines and altering the biosynthesis of nitric oxide (NO) [2], [3], [4], [5], [6]. Further, propofol inhibits neutrophil functions, including chemotaxis, attachment, migration, phagocytosis, and the production of reactive oxygen species (ROS) [2], [6]. Propofol confers antioxidant activity by scavenging free radicals and peroxynitrite to decrease oxidative stress-induced lipid peroxidation [2], [6]. As a result of these anti-inflammatory actions, the novel pharmacological effects of propofol are currently under investigation.

Intravenous propofol administration has anti-inflammatory effects *in vivo*. For example, in an endotoxemia-induced septic model, propofol inhibits stimuli-induced production of pro-inflammatory cytokines and chemokines, including tumor necrosis factor (TNF)-α, interleukin (IL)-1, IL-6, and IL-8 [2], [3], [4], [5]. Similar results have also been observed in an oleic acid-induced acute lung injury model [7].

Furthermore, propofol suppresses pro-inflammatory cytokine production and inducible NO synthase/NO biosynthesis in endotoxin lipopolysaccharide (LPS)-activated macrophages [8] and peripheral blood mononuclear cells *in vitro* [9]. Propofol also has anti-inflammatory effects on LPS-induced alveolar type II epithelial cell injury by down-regulating CD14 and toll-like receptor (TLR) 4 expression [10]. Further, propofol modulates LPS-induced inflammation in monocytic THP1 cells by inhibiting cyclooxygenase activity [11].

The molecular mechanisms for the anti-inflammatory properties of propofol have been widely investigated. In a model of polymicrobial sepsis, Song *et al.* [12] demonstrated that propofol inhibits hepatic nuclear factor (NF)-κB activation resulting in decreased production of the pro-inflammatory cytokines TNF-α and IL-6. Wu *et al.* [13] and Chiu *et al.* [14] confirmed the inhibitory effects of propofol on LPS- or lipoteichoic acid-activated NF-κB, respectively, in macrophages. Under oxidative stress-induced inflammation, propofol inhibits the phosphorylation and degradation of the inhibitor of κB (IκB) kinase (IKK) and IκB, respectively, resulting in NF-κB inactivation in hepatocytes [15]. Propofol stimulation also inhibits LPS- or lipoteichoic acid-activated mitogen-activated protein kinase (MAPK)/extracellular signal-regulated kinase (ERK), upstream regulators of NF-κB nuclear translocation [14], [16].

Infection with gram-negative bacteria causes endotoxemia-induced multiple organ failure/dysfunction syndrome or a life-threatening illness known as septic shock [17]. Severe systemic or organ inflammation contributes to the progression of sepsis; thus, the administration of anti-inflammatory agents and the promotion of anti-inflammatory processes are strategies to protect cells from LPS-induced cellular injury [18]. Inhibition of downstream LPS signaling may result in anti-inflammatory processes.

Considering the anti-inflammatory roles of propofol, we developed *in vitro* and *in vivo* approaches to investigate the protective molecular mechanisms of propofol in LPS-induced inflammatory responses in macrophages. We examined anti-inflammatory responses and signal transduction including ROS generation and the activation of Akt, MAPK/ERK1/2, and NF-κB.

## Materials and Methods

### Reagents

Propofol was prepared from Diprivan (Zeneca Limited, Macclesfield, Cheshire, UK). The vehicle contained glycerol, soybean oil, purified egg phosphatide/egg lecithin, sodium hydroxide, and water. *Escherichia coli* (*E. coli*)-derived LPS was purchased from Calbiochem (San Diego, CA, USA) and dissolved in sterile phosphate-buffered saline (PBS). NF-κB inhibitor pyrrolidine dithiocarbamate (PDTC), phosphoinositide-3 kinase (PI3K) inhibitor LY294002, PP2A inhibitor okadaic acid (OA), and antioxidant diphenylene iodonium (DPI) were obtained from Sigma-Aldrich (St. Louis, MO). They were then dissolved in DMSO prior to dilution with PBS for use in experiments. Rabbit anti-mouse iNOS, IKKβ, phospho-IKKβ (Ser180), NF-κB, phospho-NF-κB (Ser536), Akt, phospho-Akt (Ser473), p38 MAPK, phospho-p38 MAPK (Thr180/Tyr182), JNK, phospho-JNK (Thr183/Tyr185), ERK1/2, phospho-ERK1/2 (Thr185/Tyr187), PTEN, and phospho-PTEN (Ser380) were purchased from Cell Signaling Technology, Inc. (Beverly, MA, USA). β-actin antibodies and horseradish peroxidase-conjugated anti-rabbit IgG were obtained from Chemicon (Temecula, CA). All drug treatments on cells were assessed for cytotoxic effects using cytotoxicity assays prior to experiments. Non-cytotoxic dosages were used in this study.

### Animal treatment

Male C57BL/6 mice 6 weeks in age were purchased from Charles River Japan, Inc. (Atsugi, Japan). They were fed standard laboratory chow and water *ad libitum* in the Laboratory Animal Center of National Cheng Kung University. The animals were raised and cared for according to the guidelines set up by the National Science Council, Taiwan. Experimental protocols adhered to the rules of the Animal Protection Act of Taiwan and were approved by the Laboratory Animal Care and Use Committee of National Cheng Kung University (IACUC Approval No.: 99013).

To establish the endotoxemic murine model, mice (n = 3 for each group) were intraperitoneally injected with 15 mg/kg of *E. coli*-derived LPS (Calbiochem, San Diego, CA, USA) dissolved in sterile PBS; concentrations were adjusted for a total volume of 200 µL per injection. To verify the anti-inflammatory role of propofol, mice were treated with 5 mg/kg of PBS-diluted propofol in a total volume of 200 µl at the indicated time periods as previously described [3], [4], [5]. PBS was used as the vehicle control.

### Cell culture

RAW264.7 murine macrophages were provided by C-C Huang, MD, Department of Pediatrics, National Cheng Kung University. Cells were routinely grown on Petri-dishes in Dulbecco's Modified Eagle's medium (DMEM) with 2 mM L-glutamine and 15 mM HEPES supplemented with 10% fetal bovine serum (FBS), 100 units of penicillin, and 100 µg/ml of streptomycin. Cultures were kept at 37°C in an atmosphere of 5% CO_2_. Cells were used at a passage of 7 to 10 in this study.

### Viability assay

To evaluate cell viability, WST-8 assays (WST-8 Detection kit, Dojindo Molecular Technologies, Gaithersburg, MD) were performed according to the manufacturer's instructions. Cells were cultured in 96-well tissue culture plates in DMEM medium in the presence or absence of propofol. WST-8 reagent (5 µl/well) was added after 24 h of culture. A microplate reader (Spectra MAX 340PC, Molecular Devices Corporation, Sunnyvale, CA, USA) was used to measure the absorbance at 450 nm; data were analyzed with Softmax Pro software (Molecular Devices).

### Cytotoxicity assay

To evaluate cell damage, lactate dehydrogenase (LDH) activity was assayed using a colorimetric assay (Cytotoxicity Detection kit, Roche Diagnostics, Lewes, UK) performed according to the manufacturer's instructions. Aliquots of the culture media were transferred to 96-well microplates. A microplate reader (Spectra MAX 340PC, Molecular Devices) was used to measure the absorbance at 620 nm with a reference wavelength of 450 nm; data were analyzed with Softmax Pro software (Molecular Devices).

### Apoptosis assay

Apoptosis was analyzed using propidium iodide (PI) staining (Sigma Chemical Company, St Louis, MO, USA) as described previously [19]. Cells were analyzed by flow cytometry using a FACSCalibur (BD Biosciences, San Jose, CA), with excitation set at 488 nm. To observe nuclear condensation, PI-stained cells were observed using a fluorescence microscope (IX71, Olympus, Tokyo, Japan). For each test, three different and randomly selected areas were analyzed.

### Western blotting

Harvested cells were lysed with a buffer containing 1% Triton X-100, 50 mM of Tris (pH 7.5), 10 mM of EDTA, 0.02% sodium azide, and a protease-inhibitor cocktail (Roche Boehringer Mannheim Diagnostics, Mannheim, Germany). Following one freeze-thaw cycle, cell lysates were centrifuged at 10,000× *g* at 4°C for 20 min. Lysates were boiled in sample buffer for 5 min. The proteins were then subjected to SDS-PAGE and transferred to PVDF membrane (Millipore, Billerica, MA, USA) using a semi-dry electroblotting system. After blocking with 5% skim milk in PBS, the membranes were incubated with diluted primary antibodies, including phospho-IKKβ (Ser180), phospho-NF-κB (Ser536), phospho-Akt (Ser473), phospho-p38 MAPK (Thr180/Tyr182), phospho-JNK (Thr183/Tyr185), phospho-ERK1/2 (Thr185/Tyr187), phospho-PTEN (Ser380), IKKβ, NF-κB, Akt, ERK1/2, p38 MAPK, JNK, PTEN, inducible NO synthase (iNOS), and β-actin, at 4°C overnight. The membranes were then washed with 0.05% PBS-Tween 20 and incubated with a 1/5000 dilution of horseradish peroxidase-conjugated secondary antibodies at room temperature for 1 h. After washing, the membranes were soaked in ECL solution (PerkinElmer Life Sciences Inc., Boston, MA, USA) for 1 min, then exposed to film (BioMax, Eastman Kodak, Rochester, NY, USA). The relative signal intensity was quantified using ImageJ software (version 1.41o) from W. Rasband (National Institutes of Health, Bethesda, MD) (http://rsb.info.nih.gov/ij/).

### Detection of NO production

Production of NO was assessed as the accumulation of nitrite (NO_2_^−^) in the medium using a colorimetric reaction with the Griess reagent [20]. Briefly, samples (cell culture supernatants or murine ascites) were mixed with an equal (1∶1) volume of Griess reagent (0.1% *N*-(1-naphthyl) ethylenediamine dihydrochloride, 1% sulfanilamide, and 2.5% H_3_PO_4_). The absorbance was measured at 540 nm using a 96-well microplate reader (Spectra MAX 340PC, Molecular Devices); data were analyzed using Softmax Pro software. Sodium nitrite was dissolved in double-distilled water then used as standards (from 1 to 50 µM).

Enzyme-linked immunosorbent assays (ELISAs)

Cell culture supernatants and murine ascites were collected and the levels of TNF-α, IL-6, and IL-10 were measured using ELISA kits (R&D Systems, Minneapolis, MN, USA) according to the manufacturer's instructions. All samples were run in triplicate. After the reaction, plates were washed and 100 µl of *o*-phenylenediamine substrate (Sigma-Aldrich) was added to each well. Plates were incubated for 30 min at room temperature, after which, 50 µl of 4 N sulfuric acid was added to each well. The plates were read at 490 nm on a microplate reader (Spectra MAX 340PC), and the data were analyzed using Softmax Pro software.

### Immunocytochemistry staining

Cells were fixed in 3.7% formaldehyde in PBS for 10 min. After washing twice with PBS, cells were mixed with anti-NF-κB p65 antibodies (Chemicon International, Inc., Temecula, CA, USA) in antibody diluents (DAKO Corporation, Carpinteria, CA, USA), applied to the sections, and incubated at 4°C overnight. The next day, cells were washed with PBS and then incubated with Alexa Fluor 488-labeled secondary antibodies at room temperature for 1 h. Next, cells were washed with PBS and visualized under a fluorescent microscope (BX51, Olympus, Tokyo, Japan). Positive cells in three fields of each culture were quantitated.

### Intracellular ROS assay

Intracellular oxidative stress was measured by dichlorodihydrofluorescein diacetate oxidation. Cells were plated at 1×10^5^/well in 96-well plates, cultured overnight and washed twice with Hank's Buffered Salt Solution (HBSS) before experiments. Cells were exposed to 20 µM 5-(and-6)-chloromethyl-2′,7′-dichlorodihydrofluorescein diacetate, acetyl ester (CM-H_2_DCFDA) (Invitrogen Life Technologies, Carlsbad, CA, USA) for 1 h and then treated with HBSS containing the corresponding concentrations of LPS for 0.25 h either with or without propofol 0.5 h-pre-treatment. For isolated peritoneal macrophages with or without LPS treatment for 0.25 h and propofol 0.5-h pre-treatment, cells were added to HBSS containing 20 µM CM-H_2_DCFDA. Fluorescence was read immediately at wavelengths of 485 nm for excitation and 530 nm for emission on a fluorescence plate reader (Fluoroskan Ascent, Thermo Electron Corporation, Milford, MA, USA). The levels of ROS were calculated as a percentage increase compared with the control; the control was normalized to 100% of the basal level.

### Statistical analysis

Values are expressed as means ± SD. Groups were compared using Student's two-tailed unpaired *t* test or a one-way ANOVA analysis, followed by Dunnet's post-hoc test as appropriate. Statistical significance was set at p<0.05.

## Results

### Non-cytotoxic levels of propofol suppress LPS-induced iNOS/NO biosynthesis and cytokine production *in vitro* in RAW264.7 murine macrophages

**Figure 1:**
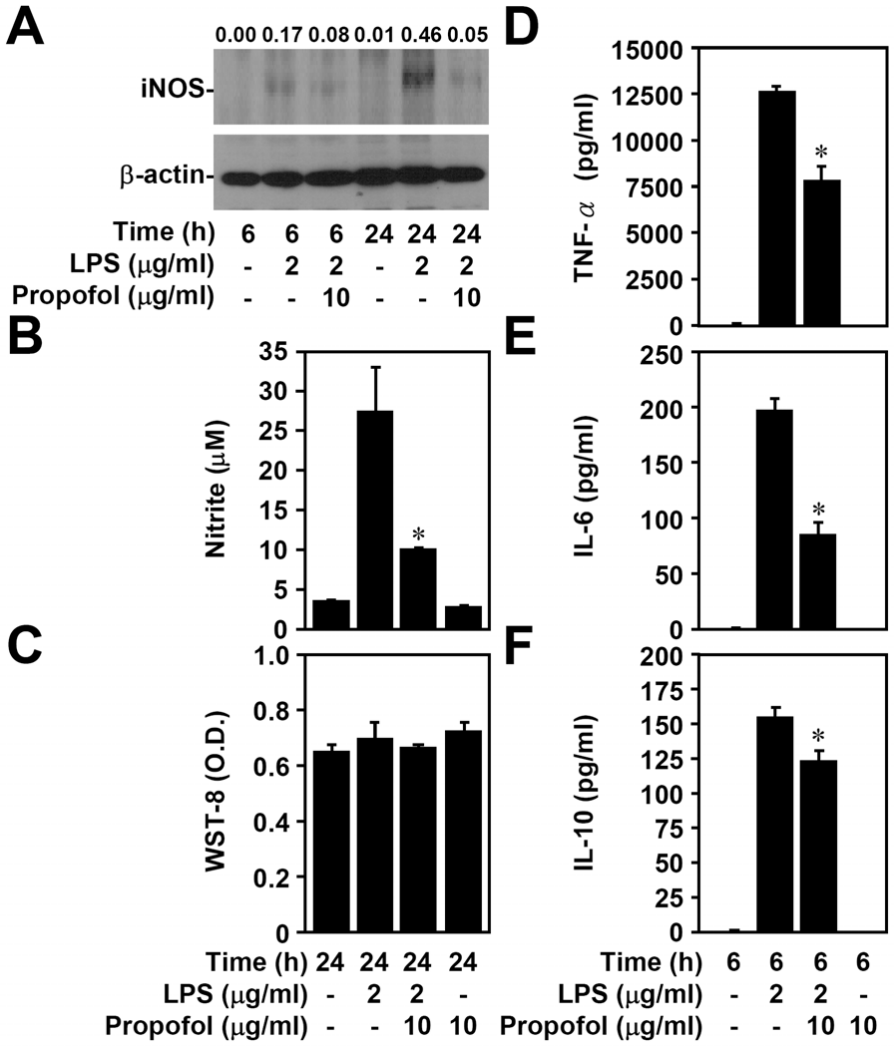
Non-cytotoxic levels of propofol reduce LPS-induced iNOS/NO biosynthesis and cytokine production. RAW264.7 cells (1×10^6^ cells/well in 6-well culture plates or 5×10^4^ cells/well in 96-well culture plates) were treated with propofol or vehicle for 0.5 h. Next, cells were stimulated with LPS (2 µg/ml) for 6 or 24 h. (A) Western blot analysis was used to determine the expression of iNOS. The ratio of iNOS to β-actin is shown; β-actin was the internal control. Data are representative of three individual experiments. (B) Griess reagent and (C) WST-8 were used to detect the generation of nitrite and cytotoxicity, respectively. Levels of TNF-α (D), IL-6 (E), and IL-10 (F) in culture supernatants were determined by ELISA. Data, obtained from triplicate cultures, are means ± SD. One of representative data obtained from three individual experiments is shown. *p<0.05 compared to the LPS group.

To avoid any cytotoxic effects caused by propofol, we investigated the effects of propofol on cell survival and cytotoxicity in RAW264.7 murine macrophages. Viability and cytotoxicity were assessed using WST-8 and LDH assays; these results showed that treatment with 10 µg/ml of propofol did not cause RAW264.7 cell death (data not shown). LPS stimulation typically induces inflammatory responses such as iNOS/NO biosynthesis and increased production of pro-inflammatory cytokines in macrophages [20]. To investigate the anti-inflammatory effects of propofol, we used western blotting and the Griess reaction, respectively, to determine the expression of iNOS and nitrite, as indicators for NO generation. We found that pre-treatment with propofol (10 µg/ml) significantly (p<0.05) reduced LPS-upregulated iNOS (0.46 with LPS only vs. 0.05 with LPS + propofol, Figure 1A) and nitrite (27.1±6.9 with LPS only vs. 9.4±0.2 with LPS + propofol, Figure 1B) 24 h after LPS treatment. To confirm that cytoxicity was not influencing our findings, WST-8 analysis was performed at the 24 h-post-treatment time point; results did not show any evidence of cytotoxicity (Figure 1C). We also used ELISAs to measure production of the cytokines TNF-α, IL-6, and IL-10 from LPS-treated RAW264.7 macrophages. We found that pre-treatment with propofol significantly (p<0.05) reduced LPS-induced upregulation of TNF-α (12513.2±297.6 with LPS only vs. 7583.1±1025.2 with LPS + propofol, Figure 1D), IL-6 (192.1±12.8 with LPS only vs. 88.5±13.7 with LPS + propofol, Figure 1E), and IL-10 (153.6±7.1 with LPS only vs. 120.9±6.8 with LPS + propofol, Figure 1F) *in vitro*. These results show that non-cytotoxic levels of propofol suppress LPS-induced inflammatory responses in macrophages as measured by iNOS/NO biosynthesis and cytokine production.

### Non-cytotoxic levels of propofol reduce LPS-induced activation of NF-κB *in vitro*

**Figure 2:**
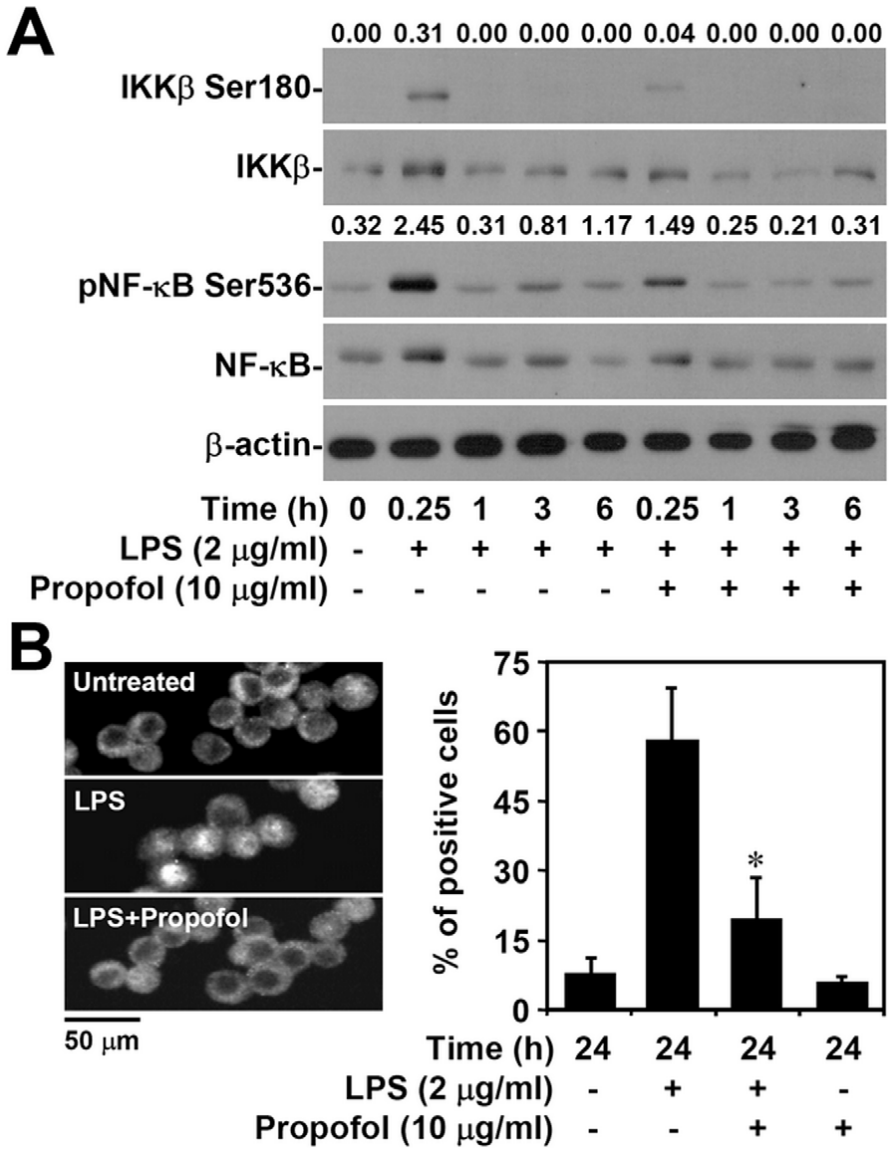
Non-cytotoxic levels of propofol inhibit LPS-induced NF-κB activation. RAW264.7 cells (1×10^6^ cells/well in 6-well culture plates or 5×10^4^ cells/well in 96-well culture plates) were treated with propofol or vehicle for 0.5 h. Next, cells were stimulated with LPS (2 µg/ml) for 6 or 24 h. (A) Western blot analysis was used to determine the phosphorylation of IKKβ (Ser180) and NF-κB (Ser536). β-actin was the internal control. The ratios of pIKKβ to IKKβ and pNF-κB to NF-κB are shown, respectively. Data are representative of three individual experiments. (B) After 0.25-h post-treatment, fluorescence microscopy was used to determine the nuclear translocation of NF-κB p65 in RAW264.7 cells (5×10^4^ cells/well in 96-well culture plates) immunostained with anti-NF-κB p65 antibody. Scale bar is 50 µm. Data obtained from three different areas are means ± SD. One of representative data obtained from three individual experiments is shown. *p<0.05 compared with the LPS group.

Propofol may act upstream of NF-κB [13], [14], [16], an important transcription factor regulating iNOS and TNF-α production. Utilizing western blots, we found that propofol treatment reduced LPS-induced phosphorylation of IKKβ (Ser180) (0.31 with LPS only vs. 0.04 with LPS + propofol), which is an important upstream kinase for IκB degradation and subsequent NF-κB activation [21], [22]. Further, phosphorylation of NF-κB (Ser536) was reduced after 0.25 h of LPS treatment (2.45 with LPS only vs. 1.49 with LPS + propofol, Figure 2A). To further investigate the effect of propofol on NF-κB signaling, we used immunocytochemistry to examine the nuclear translocation of NF-κB p65. We found that treatment with propofol significantly (p<0.05) inhibited LPS-induced NF-κB p65 nuclear translocation (56.7±11.3 with LPS only vs. 17.7±10.1 with LPS + propofol, Figure 2B). To confirm the essential role of NF-κB in LPS-induced inflammatory responses of macrophages, we pre-treated macrophages with the NF-κB inhibitor pyrrolidine dithiocarbamate; pre-treatment significantly reduced LPS-induced upregulation of nitrite (data not shown). Taken together, these results show that propofol treatment reduces LPS-induced inflammatory responses in macrophages primarily by inhibiting NF-κB activation.

### Non-cytotoxic levels of propofol reduce LPS-induced activation of Akt *in vitro*

**Figure 3:**
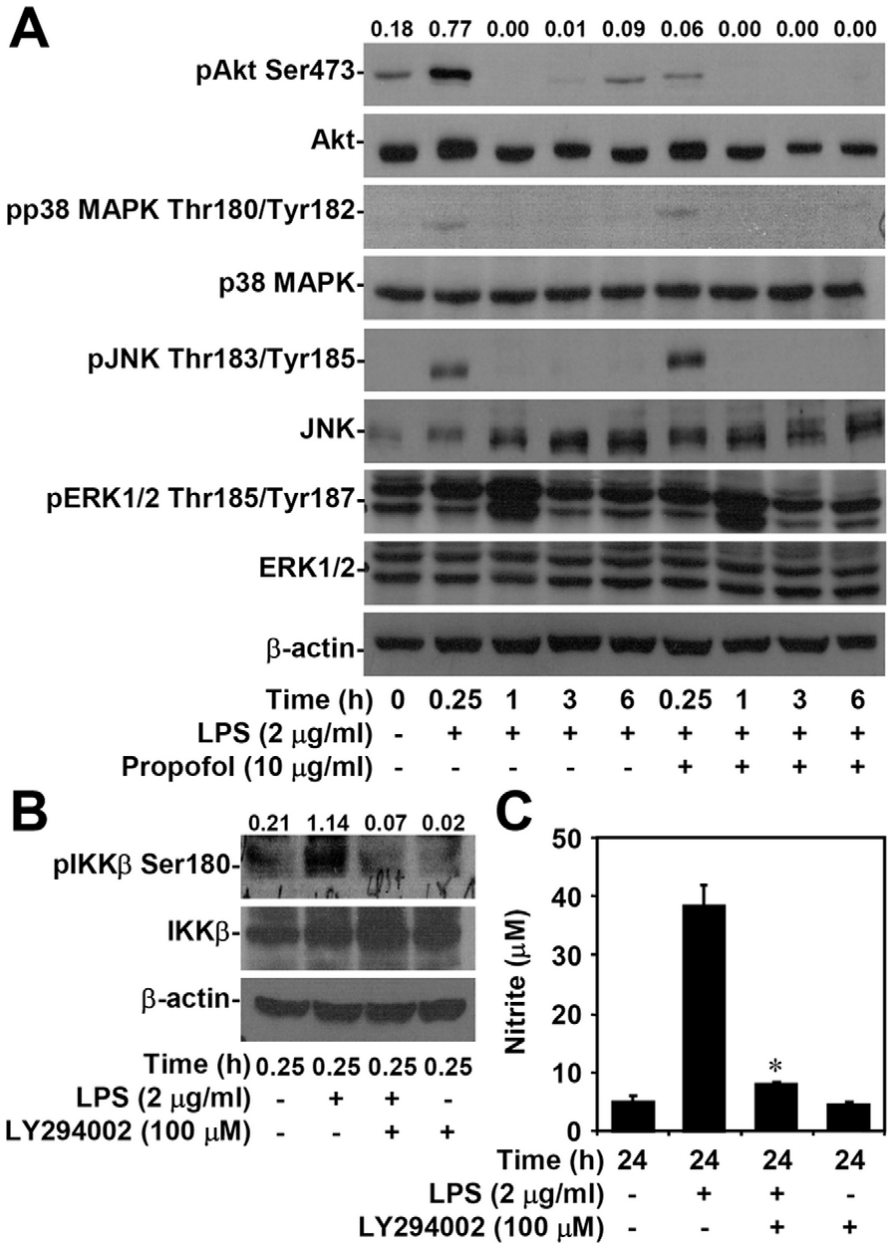
Non-cytotoxic levels of propofol inhibit LPS-induced Akt activation, which Akt signaling is required for LPS-induced NF-κB activation as well as NO generation. RAW264.7 cells (1×10^6^ cells/well in 6-well culture plates or 5×10^4^ cells/well in 96-well culture plates) were treated with propofol or vehicle for 0.5 h. Next, cells were stimulated with LPS (2 µg/ml) for 6 or 24 h. (A) Western blot analysis was used to determine the phosphorylation of Akt (Ser473), p38 MAPK (Thr180/Tyr182), JNK (Thr183/Tyr185), and ERK1/2 (Thr185/Tyr187). β-actin was the internal control. The ratio of pAkt to Akt is shown. Data are representative of three individual experiments. (B) RAW264.7 cells (1×10^6^ cells/well in 6-well culture plates) were treated with LPS (2 µg/ml) for the indicated time periods with or without LY294002 (100 µM) pre-treatment for 0.5 h. Western blot analysis was used to determine the phosphorylation of IKKβ (Ser180). β-actin was the internal control. The ratio of pIKKβ to IKKβ is shown. Data are representative of three individual experiments. (C) Meanwhile, Griess reagent was used to detect the generation of nitrite. Data, obtained from triplicate cultures, are means ± SD. One of representative data obtained from three individual experiments is shown. *p<0.05 compared to the LPS group.

Activation of MAPKs and Akt may act upstream of NF-κB signaling [22], [23], [24], [25], [26]. We found that propofol treatment reduced LPS-induced phosphorylation of Akt (Ser473) (0.77 with LPS only vs. 0.06 with LPS + propofol) but not ERK1/2 (Thr185/Tyr187), p38 MAPK (Thr180/Tyr182), or JNK (Thr183/Tyr185) 0.25 h after LPS treatment (Figure 3A). To confirm the effect of Akt on NF-κB activation, we demonstrated that LY294002, a PI3K inhibitor, reduced LPS-induced phosphorylation of IKKβ (Ser180) 0.25 h after LPS treatment (1.14 with LPS only vs. 0.07 with LPS + LY294002, Figure 3B). We further found that pre-treatment with LY294002 significantly (p<0.05) reduced LPS-induced upregulation of nitrite in macrophages *in vitro* (37.8±4.9 with LPS only vs. 7.4±0.3 with LPS + propofol, Figure 3C). Overall, these results demonstrate that treatment with propofol reduces LPS-induced inflammatory responses in macrophages by inhibiting Akt phosphorylation and Akt-regulated NF-κB activation.

### Non-cytotoxic levels of propofol reduce LPS-induced ROS generation *in vitro*

**Figure 4:**
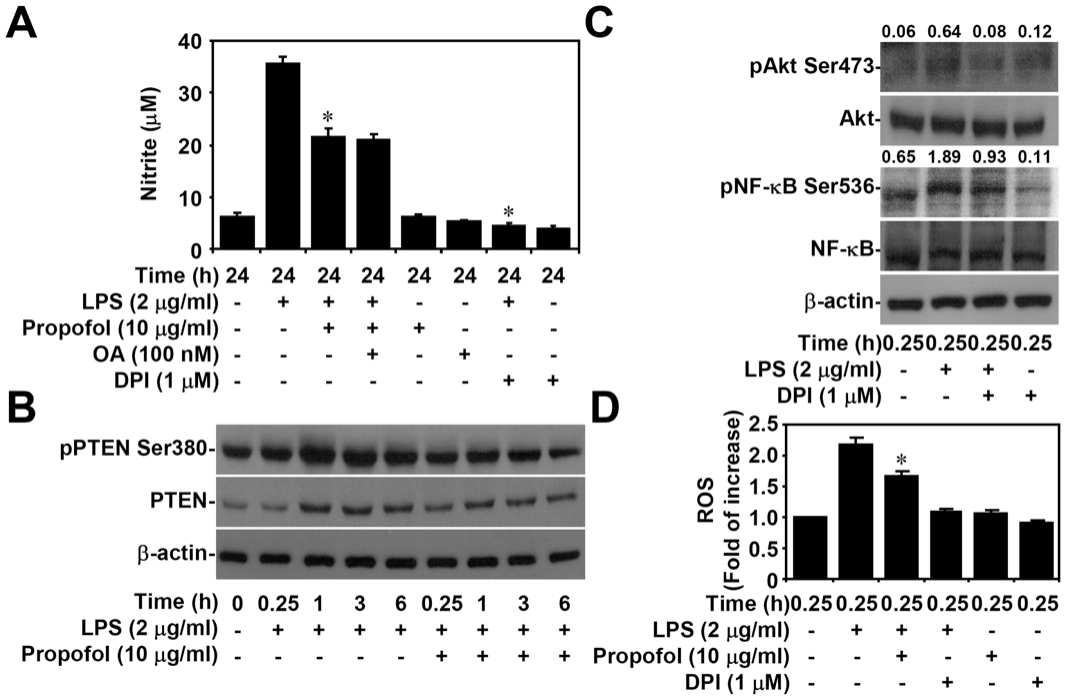
Non-cytotoxic levels of propofol decrease LPS-induced ROS generation, which ROS is required for LPS-induced activation of Akt and NF-κB as well as NO generation. RAW264.7 cells (5×10^4^ cells/well in 96-well culture plates) were treated with LPS (2 µg/ml) for 24 h with or without propofol (10 µg/ml), okadaic acid (100 nM), or DPI (1 µM) pre-treatment for 0.5 h. (A) Griess reagent was used to detect the generation of nitrite. Data, obtained from triplicate cultures, are means ± SD. One of representative data obtained from three individual experiments is shown. *p<0.05 compared to the LPS group. (B and C) RAW264.7 cells (1×10^6^ cells/well in 6-well culture plates) were treated with LPS (2 µg/ml) for the indicated time periods with or without propofol (10 µg/ml) or DPI (1 µM) pre-treatment for 0.5 h. Western blot analysis was used to determine the phosphorylation of PTEN (Ser380), Akt (Ser473), and NF-κB (Ser536). β-actin was the internal control. The ratios of pAkt to Akt and pNF-κB to NF-κB are shown, respectively. Data are representative of three individual experiments. (D) RAW264.7 cells (5×10^4^ cells/well in 96-well culture plates) were treated with LPS (2 µg/ml) for 0.25 h with or without propofol (10 µg/ml) or DPI (1 µM) pre-treatment for 0.5 h. CM-H_2_DCFDA was used to determine the generation of intracellular ROS. Data, obtained from triplicate cultures as shown as fold of increase, are means ± SD. One of representative data obtained from three individual experiments is shown. *p<0.05 compared to the LPS group.

Protein phosphatases (PPases) such as PP2A and PTEN are negative regulators for Akt signaling [19]. Pre-treatment with the PP2A inhibitor okadaic acid (OA) did not reverse the ability of propofol to inhibit LPS-induced upregulation of nitrite in macrophages (20.6±1.9 without OA vs. 20.1±1.1 with OA, Figure 4A). Furthermore, propofol treatment did not increase LPS-induced phosphorylation and subsequent activation of PTEN (Ser380) (Figure 4B). These results indicate that the mechanism used by propofol to inhibit Akt is independent of PP2A and PTEN.

Current studies have shown that propofol acts as antioxidant to downregulate oxidative stress [2]. As ROS are critical for LPS-induced inflammation through activation of Akt as well as NF-κB signaling [24], [27], [28], we further investigated the effects of propofol on LPS-induced ROS signaling. First, treatment with the antioxidant DPI significantly (p<0.05) reduced LPS-induced upregulation of nitrite (Figure 4A), suggesting the essential role of ROS in LPS-induced inflammatory responses. Western blot analysis demonstrated that DPI reduced LPS-induced phosphorylation of Akt (Ser473) (0.64 with LPS only vs. 0.08 with LPS + DPI) and phosphorylation of NF-κB (Ser536) (1.89 with LPS only vs. 0.93 with LPS + DPI) 0.25 h after LPS treatment (Figure 4C). To examine the effect of propofol on ROS, we used CM-H_2_DCFDA staining to demonstrate that propofol significantly (p<0.05) reduced LPS-induced upregulation of ROS *in vitro* (2.2±0.2 with LPS only vs. 1.6±0.1 with LPS + propofol, Figure 4D). Taken as a whole, these results show that propofol reduces LPS-induced inflammatory responses in macrophages partly by inhibiting ROS and ROS-regulated Akt and NF-κB activation.

### Non-cytotoxic levels of propofol inhibit LPS-induced ROS generation, NF-κB activation, and inflammation *in vivo*

**Figure 5:**
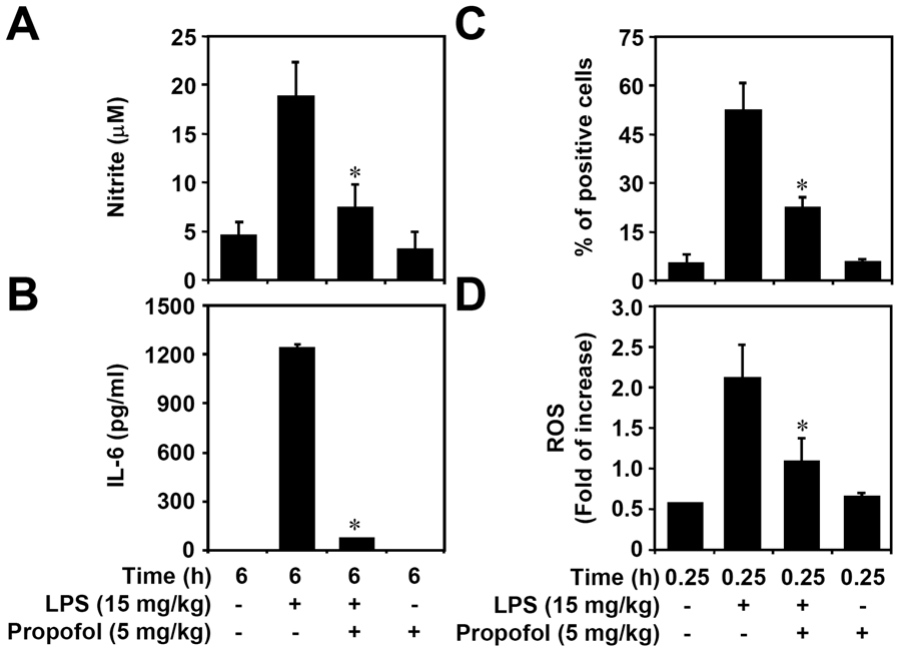
Non-cytotoxic levels of propofol reduce LPS-induced inflammation *in vivo*. C57BL/6 (n = 3) mice were intraperitoneally injected with LPS (15 mg/kg) with or without propofol (5 mg/kg) pre-treatment for 0.5 h. At the indicated time periods, mice were sacrificed and their peritoneal macrophages and ascites were isolated. (A and B) Ascites levels of nitrite and IL-6 were determined by the Griess reaction and ELISA, respectively. Data, obtained from three mice, are means ± SD. One of representative data obtained from three individual experiments is shown. *p<0.05 compared to the LPS group. (C) Fluorescence microscopy was used to determine the nuclear translocation of NF-κB p65 in peritoneal macrophages immunostained with anti-NF-κB p65 antibody. Data, obtained from three mice, are means ± SD. One of representative data obtained from three individual experiments is shown. *p<0.05 compared to the LPS group. (D) CM-H_2_DCFDA was used to determine the generation of intracellular ROS in isolated peritoneal macrophages. Data, obtained from three mice, are means ± SD and these experiments were confirmed by independent repetitions. *p<0.05 compared to the LPS group.

To investigate the anti-inflammatory effects of propofol *in vivo*, we used the Griess reaction and ELISA, respectively, to determine the *in vivo* production of nitrite and IL-6 in LPS-treated (15 mg/kg) C57BL/6 mice. We found that pre-treatment with propofol (5 mg/kg) significantly (p<0.05) reduced LPS-induced upregulation of nitrite (18.1±4.0 with LPS only vs. 6.9±3.1 with LPS + propofol, Figure 5A) and IL-6 (1209.2±25.8 with LPS only vs. 50.7±7.6 with LPS + propofol, Figure 5B) in the ascites of treated mice. Western blot analysis demonstrated that propofol reduced LPS-induced phosphorylation of Akt (Ser473) and NF-κB (Ser536) (data not shown) in isolated peritoneal macrophages. To further investigate the effect of propofol on NF-κB signaling, we used immunocytochemistry to examine the nuclear translocation of NF-κB p65 in isolated peritoneal macrophages. We found that propofol treatment significantly (p<0.05) reduced LPS-induced NF-κB p65 nuclear translocation (47.3±10.2 with LPS only vs. 17.1±4.5 with LPS + propofol, Figure 5C). Notably, utilizing CM-H_2_DCFDA staining, we found that propofol significantly (p<0.05) reduced LPS-induced generation of ROS (2.1±0.5 with LPS only vs. 1.1±0.3 with LPS + propofol, Figure 5D). These results show that propofol suppresses LPS-induced inflammatory activation *in vivo* in peritoneal macrophages partly by inhibiting LPS-induced activation of NF-κB as well as ROS generation.

## Discussion

Anesthetic propofol has been shown to possess anti-inflammatory properties. Propofol can suppress cytokine and chemokine production and iNOS/NO biosynthesis and inhibit the generation of inflammatory mediators, both *in vivo* and *in vitro*. However, the molecular mechanisms responsible for the anti-inflammatory actions of propofol remain unclear. Recent studies [12], [14], [15], [16] have been focused on propofol's inhibitory activities against LPS- or inflammatory stimuli-induced signal transduction, particularly targeting the NF-κB pathway. These studies [14], [16] successfully identified potential actions for propofol-mediated inhibitory signaling through modulation of MAPK/ERK1/2, which acts upstream of NF-κB signaling. However, whether these targets are affected by propofol through direct or indirect regulation still remains unclear. In the present study, we developed *in vitro* and *in vivo* approaches to examine LPS/TLR4-mediated inflammation characterized by iNOS/NO biosynthesis and cytokine production in macrophages. We showed that propofol treatment reduced LPS-induced cellular inflammatory responses. Furthermore, treatment with propofol suppressed LPS-activated NF-κB signaling by inhibiting phosphorylation of IKKβ (Ser180) and NF-κB (Ser536) and the subsequent nuclear translocation of NF-κB. Notably, propofol treatment reduced ROS generation and ROS-mediated Akt activation, which are critical mediators in NF-κB activation. We hypothesize that propofol inhibits LPS-induced inflammatory responses in macrophages partly through the mechanisms of ROS, Akt, and NF-κB inactivation.

The anti-inflammatory properties of non-cytotoxic levels of propofol (lower than 10 µg/ml) on LPS-activated RAW264.7 macrophages were demonstrated in this study. However, abusive treatment with propofol can cause severe complications in patients with critical illnesses, so-called propofol infusion syndrome (PRIS) [29], [30]. Clinical manifestations and pathological observations showed a variety of cellular injury in PRIS patients, including lipemic plasma, fatty liver enlargement, metabolic acidosis, rhabdomyolysis, and myoglobinuria. In regard to the immune system, an overdose of propofol has been shown to cause the loss of circulating leukocytes in an experimental animal model [31], impair immune responses and increase susceptibility to severe infection [29]. We showed that treatment with a high dosage of propofol (25 µg/ml) resulted in macrophage apoptosis (data not shown). In PRIS patients, we hypothesize that propofol may cause immunosuppression not only through inflammatory inactivation by inhibiting ROS and the Akt and NF-κB signaling pathways but also through the induction of cell apoptosis. This hypothesis and mechanism are currently under investigation.

Consistent with previous studies [13], [14], [16], we showed that propofol suppressed LPS-induced phosphorylation of IKKβ (Ser180) and NF-κB (Ser536) and inhibited subsequent NF-κB activation *in vitro* in RAW264.7 macrophages; similar results were observed in peritoneal macrophages in an *in vivo* model. These results indicate that propofol may inhibit LPS/TLR4-activated NF-κB signaling and inflammatory responses. Although MAPKs are involved in LPS-induced inflammation in RAW264.7 macrophages [22], [23], [24], [25], [26], we demonstrated that LPS-activated Akt was inhibited by propofol and that propofol treatment did not affect MAPKs, including ERK1/2, p38 MAPK, and JNK. Previously, our work as well as others [23], [26] demonstrated that LPS-activated Akt was critical for NF-κB-mediated inflammatory responses in macrophages. However, this finding is inconsistent with previous studies that found that propofol reduces MAPK/ERK1/2 signaling to downregulate NF-κB in LPS-activated hepatocytes [16] and lipoteichoic acid-activated macrophages [14]. It is speculated that the different effects caused by propofol are dependent upon cell type and type of stimulation; further investigation is required.

The antioxidant activity of propofol has been previously reported [2], and it is known to exert important pharmacological effects on anti-inflammation. ROS are critical for NF-κB activation [24], [27], [28] and Akt activation [24] in LPS/TLR4 signaling. To clarify the causes for propofol-induced inactivation of Akt, we demonstrated, for the first time, propofol-mediated Akt and NF-κB inactivation partly through ROS downregulation. This action was independent of the activation of protein phosphatases such as PP2A or PTEN. Our findings suggest that antioxidant activity is the key for propofol-mediated Akt and NF-κB inactivation in LPS-activated RAW264.7 macrophages. We hypothesize that this mechanism, in addition to the previously reported inhibition of the MAPK/ERK1/2 pathway [14], [16], is responsible for the immunomodulatory effects of propofol on LPS-activated macrophages.

In an experimental endotoxemic animal model, combined treatment with propofol and dexamethasone reduced mortality rate and attenuated organ injury [32]. These protective effects may be associated with their anti-inflammatory capacity and antioxidant activity. An antiseptic effect of propofol is therefore speculated and needs further investigation because of endotoxemic sepsis using the animal models is poorly consistent with clinical features of human sepsis [33], [34]. Limitations including aging, types of animal, treatment protocol, doses, the timing periods of administration, and septic inducers are critical for evaluating the therapeutic effects of drugs. Studies on the molecular targets and actions of propofol are important for exploring its further pharmacological effects for the benefit of patients. Placing our work in context with previous findings [13], [16], we hypothesize that propofol acts as an anti-inflammatory agent that suppresses LPS/TLR4-mediated inflammation through the inhibition of NF-κB activation in macrophages. Basically, oxidative stress contributes to septic inflammation and cellular injury by causing activation of inflammatory mediators, including ROS, transcription factors, and MAPKs, and dysfunction of survival-associated proteins, lipids, and DNA [35], [36]. We and others [24], [27], [28] showed that ROS regulate Akt as well as NF-κB signaling while activation of MAPKs and Akt may act upstream of NF-κB [22], [23], [24], [25], [26]. Antioxidants such as selenium, glutamine, omega-3 fatty acid, melatonin, and vitamin C are widely utilized to prevent the progression of sepsis by inhibiting oxidative inflammation as well as cellular injury [37], [38]. We further provide evidence that propofol exhibits antioxidant activity capable of regulating ROS-mediated Akt and NF-κB signaling *in vitro* and *in vivo*. These results indicate a novel pharmacological action by propofol for anti-oxidation and anti-inflammation in the future.
